# Coevolution of Myoelectric Hand Control under the Tactile Interaction among Fingers and Objects

**DOI:** 10.34133/2022/9861875

**Published:** 2022-11-16

**Authors:** Yuki Kuroda, Yusuke Yamanoi, Shunta Togo, Yinlai Jiang, Hiroshi Yokoi

**Affiliations:** ^1^Joint Doctoral Program for Sustainability Research, Graduate School of Informatics and Engineering, The University of Electro-Communications, Tokyo, Japan; ^2^Department of Mechanical and Intelligent System Engineering, Graduate School of Informatics and Engineering, The University of Electro-Communications, Tokyo, Japan; ^3^Center for Neuroscience and Biomedical Engineering, The University of Electro-Communications, Tokyo, Japan; ^4^Beijing Innovation Center for Intelligent Robots and Systems, Beijing, China

## Abstract

The usability of a prosthetic hand differs significantly from that of a real hand. Moreover, the complexity of manipulation increases as the number of degrees of freedom to be controlled increases, making manipulation with biological signals extremely difficult. To overcome this problem, users need to select a grasping posture that is adaptive to the object and a stable grasping method that prevents the object from falling. In previous studies, these have been left to the operating skills of the user, which is extremely difficult to achieve. In this study, we demonstrate how stable and adaptive grasping can be achieved according to the object regardless of the user's operation technique. The required grasping technique is achieved by determining the correlation between the motor output and each sensor through the interaction between the prosthetic hand and the surrounding stimuli, such as myoelectricity, sense of touch, and grasping objects. The agents of the 16-DOF robot hand were trained with the myoelectric signals of six participants, including one child with a congenital forearm deficiency. Consequently, each agent could open and close the hand in response to the myoelectric stimuli and could accomplish the object pickup task. For the tasks, the agents successfully identified grasping patterns suitable for practical and stable positioning of the objects. In addition, the agents were able to pick up the object in a similar posture regardless of the participant, suggesting that the hand was optimized by evolutionary computation to a posture that prevents the object from being dropped.

## 1. Introduction

People with upper limb deficiencies desire a prosthetic hand with the same dexterity as a human hand [1] due to the reduced quality of life caused by the loss of hand function. Therefore, developing a dexterous five-fingered prosthetic hand that can be intuitively controlled by biological signals is primary goal of researchers in this field.

The complexity of manipulating a prosthetic hand using biological signals increases significantly with an increase in the degrees of freedom. Although humans can intuitively perform adaptive grasping, users of prosthetic hands cannot perform it easily. However, conventionally, prosthetic hand users are expected to develop the skills to perform effective manipulation.

In this section, we will introduce previous research that has been done to realize dexterous prosthetic hand control that solves these problems and clarify the forgotten problems.

Myoelectricity, an electrical signal generated when a muscle contracts, has been used to control electric prosthetic hands since their advent. This is because most of the muscles that drive the hand are located in the forearm, and the motion intention can be easily extracted from the myoelectricity of the residual limb. To realize multi-degree-of-freedom control from myoelectricity, a control method based on pattern recognition has been widely studied [2–5]. The pattern recognition method refers to a supervised learning method to classify the motion intention from myoelectric data based on pregiven teacher data, which involve pairs of motion intention and myoelectric data. It has been believed that the relationship between myoelectric patterns and prosthetic hand movements is a one-to-one correspondence, and if the user can master multiple myoelectric patterns, multi-degree-of-freedom control will be possible.

However, this method requires the user to select a grasping posture adapted to the object, which is typically performed subconsciously by humans, resulting in reduced operability. In other words, for dexterous manipulation (software) of the mechanical elements of the fingers (hardware), the integration of these two elements must be studied. However, research on the interpolation of human adaptive grasping functions has been conducted independently in software and hardware.

On the hardware side, adaptive grasping with underactuated hands has been studied [6–9]. In particular, Fukaya et al. realized 11 out of 14 grasping classifications specified by Kamakura [10] using a robotic hand with a cooperative link mechanism [9]. In this framework, links connect each finger, and the power from one motor is branched and transmitted to the five fingers. Consequently, each finger automatically maintains the balance of the contact force. When this mechanism is applied to a myoelectric prosthetic hand (MPH), the MPH can adaptively grasp the object according to the object shape through a simple electromyogram (EMG) and few motors. However, while the underactuated hand can adapt to the object and grasp it, it has the disadvantage that it cannot precisely manipulate the object, or gesture without interacting with the object, which limits the hardware.

On the software side, methods for optimizing the grasping motion combined with image recognition have been studied [11, 12]. For example, Ghazaei et al. used a built-in web camera to predetermine the grasping motion based on the images obtained before grasping, which enabled the realization of four grasping motions according to the object shape [12]. However, the developer predetermined the correspondence between the object shape and the grasping motion only from the image data. In reality, the grasping motion cannot be determined sufficiently from the image data only because the size and orientation of the same type of object may be different. Therefore, to control the hand as if it were a real hand, it is necessary to determine the hand motion by considering the interaction between the object and the hand.

Because of the limitations of the hardware approach as mentioned above, it is necessary to use a software approach to achieve adaptive grasping of objects. However, in previous studies, the problem has been solved without considering the interaction between the object to be grasped and the hand, and thus, a satisfactory solution has not been obtained.

Considering these aspects, this study was aimed at developing an MPH control method that can realize dexterous multi-degree-of-freedom (DOF) control from a small number of EMG patterns. We focused on object grasping motion, which is the most commonly used motion in daily life. Pfeifer and Bongard highlighted that informative and correlated sensory signals are generated in different sensory channels through an agent's physical interaction with its environment [13]. Based on this concept, considering the interaction between the MPH and surrounding environment (myoelectricity, tactile sensation, grasped object, etc.), the correlation between the motor output and each sensor can be obtained, and adaptive and stable grasping is realized according to the object.

Such a control method can be developed using evolutionary robotics, which can help automatically generate artificial brains and morphologies of autonomous robots [14]. According to physical simulations, this method can be used to pick up objects with a robot arm [15] and identify the grasped objects [16, 17]. In general, this method is implemented by training a controller composed of a recurrent neural network (RNN) through evolutionary computation. However, none of the existing research methods on object grasping based on evolutionary robotics can control the open/close states of the hand via myoelectricity.

In this study, by adapting the evolutionary robotics approach to the control of a myoelectric prosthetic hand, adaptive grasping was realized via myoelectricity by predicting the appropriate joint motion based on the information of the contact state between the hand and grasped object, as obtained from a touch sensor. Since it is challenging to prepare a fully actuated robot hand with multiple degrees of freedom, we use physical simulation to verify the effectiveness of our approach. Because the necessary elements of this method are joint control and haptic feedback, this method can be fully applied in real environments.

We evaluated the agents of a 16-DOF robot hand trained with myoelectric signals from six participants, including one child with congenital forearm deficiency. Consequently, the agents of all participants were able to accomplish the pickup task. In addition, regardless of the participant, the agents performed the pickup task in a similar posture to the object, suggesting that the hand was optimized by evolutionary computation to a posture that prevents the object from being dropped. In the grasping experiment for one of the agents with different object shapes and sizes, the agent was able to pick up objects of unlearned shapes and sizes. These results indicate that the proposed method can adaptively grasp objects with simple myoelectric control using a multi-degree-of-freedom myoelectric prosthetic hand, regardless of the type of user.

## 2. Proposed Method: Learning Myoelectric Control and Adaptive Object Grasping via Evolutionary Computation

In the conventional control method of the MPH, different muscle contraction patterns must be used for different hand motions. However, as mentioned in Section 1, this aspect presents a burden for people with upper limb deficiencies. In particular, it is challenging to select a grasping posture that is adaptive to the object and a stable grasping method that does not drop the object. Through an existing approach to realize object grasping through evolutionary robotics, various types of objects can be grasped and classified using a robot hand. In this framework, the robot hand and object to be grasped interact with each other, and the input from the touch sensor on the robot hand is mapped to the output from the motor that controls the joints of the robot hand. This mechanism is based on the concept of embodiment proposed by Pfeifer and Bongard [13]. However, none of the existing research methods pertaining to object grasping based on evolutionary robotics can control the open/close state of the hand through myoelectricity.

If adaptive grasping can be realized by predicting the appropriate scheme to move the joints—based on the information of the contact state between the hand and grasped object, as obtained from the touch sensor—and we can control the voluntary opening and closing of the joints via the EMG, we can achieve appropriate grasping of various objects even with a simple EMG input. With reference to the existing research, the joint movements are predicted by making the robot hand repeatedly perform object pickup tasks and optimizing the parameters of the robot hand controller to enhance the performance in the task through evolutionary computation. Notably, in addition to the conventional pickup learning environment for autonomous robots, we add an environment for MPH control learning by using the teacher data of grasping and opening motions. In this manner, we can establish an MPH control method that can open and close the hand at arbitrarily while enabling the adaptive grasping of objects.

**Figure 1:**
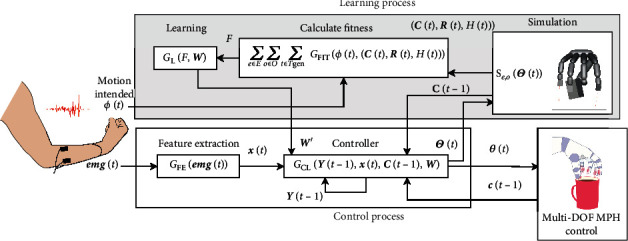
Overview of the proposed method. **x**(*t*) is the feature vector at time *t*. **C**(*t*) is the set of the individual vectors of touch sensor inputs **c**(*t*). **R**(*t*) is the set of vectors of distance sensor inputs for each individual. *H*(*t*) is the set of height sensor inputs for each individual. **W** is the set of parameter vector for the controllers of each individual. Θ(*t*) is the set of target joint angle vectors Θ(*t*) for controlling the MPH. *E* is the set of environments *e* of the simulation. *O* is the set of objects *o* used in the simulation. *T*_gen_ is the number of the steps for which the simulation is performed. *F* is the set of fitness values calculated using the fitness calculation function *G*_FIT_ for each individual. **W**′ is the set of the parameter vectors updated from **W**.

An overview of the proposed method is shown in Figure 1. Evolutionary computation is performed to optimize the parameters in parallel. Therefore, let *P* be the number of individuals in the population; the sets of vectors and scalars are represented as capital letters that are boldfaced and not boldfaced (e.g., **W** = (**w**_1_, **w**_2_, ⋯**w**_*p*_, ⋯, **w**_*P*_)), respectively. In the case of a variable, a vector, or a set of variables in the *t*-th step, the parameter is denoted as (*t*) (e.g., **x**(t)). All the considered sets represent the population.

This method involves two processes: (a) learning process and (b) control process.

In the control process, after measuring **emg**(*t*), which is the EMG vector measured at time *t*, feature vector **x**(*t*) is output by applying **emg**(*t*) to the feature extraction function *G*_FE_. When the *p*-th individual exhibits the highest fitness after the learning process, the controller yields the vector of the target joint angles **θ**_*p*_(*t*) by calculating the vector of the neurons of the *p*-th controller **y**_*p*_(*t* − 1), **x**(*t*), vector of touch sensors **c**(*t* − 1), and parameters for the *p*-th controller **w**_*p*_. In the learning process, the controller *G*_*L*_ outputs the set of target vectors for the individuals by calculating the set of vectors of neurons, **Y**(*t* − 1), **x**(*t*), set of vectors of touch sensors for the individuals, **C**(*t* − 1), and set of the vector of parameters for the controllers, **W**. The fitness calculation function *G*_FIT_ receives the motion intent *ϕ*(*t*), **C**(*t*), set of vectors of distance sensors, **R**(*t*), and set of height sensors, *H*(*t*), from *S*_*e*,*o*_ for each step. After the simulation of the *T*_gen_ step is completed for all environments *E* and objects *O*, the set of fitness values *F* is obtained. The learning function *G*_L_ updates **W** based on *F*. This section describes the morphology, sensory organs, controllers, learning methods, fitness functions, and tasks for the robot hand.

### 2.1. Morphology

We train the MPH control system by using 3D models on a physics simulator. The shapes of the 3D models used in the evaluation experiments are described in Section 3.

### 2.2. Sensory Organs

#### 2.2.1. EMG Sensor

Let **emg**(*t*) = (*emg*(*t* − *T*_EMG_),  *emg*(*t* − (*T*_EMG_ − 1)), ⋯,  *emg*(*t* − 1),  *emg*(*t*)) be the vector of muscle potentials measured from the EMG sensor of a user. Next, we translate this vector by applying the feature extraction function *G*_FE_, as shown.

(1) $$\begin{array}{c} x\left(t\right)=G_{\text{FE}}\left(emg\left(t\right)\right). \end{array}$$

#### 2.2.2. Touch Sensor

The hands are equipped with touch sensors. The input of the *i*-th touch sensor to the controller *c*_*i*_(*t*) can be defined as follows:

(2) $$\begin{array}{c} c_{i}\left(t\right)=\left\{\begin{array}{cc} 1. & \text{if the part touches the floor or the objects},\\ 0. & \text{otherwise}. \end{array}\right. \end{array}$$

Here, let **C**(*t*) = (**c**_1_(*t*), ⋯, **c**_*p*_(*t*), ⋯, **c**_|*C*(*t*)|_(*t*)) be the set of individuals in *c*_*p*_(*t*) ∈ ℝ^*N*_*c*_^, which is the vector of the touch sensor inputs to the *p*-th controller. The placement of and number of sensors are described in Section 3.

#### 2.2.3. Distance Sensor

Distance sensors are installed in each fingertip of the 3D model to measure the distance to an object when the ray of light from the fingertip strikes it. The value of the *i*-th distance sensor when the number of steps is *t* can be determined as

(3) $$\begin{array}{c} r_{i}\left(t\right)=1-\frac{d_{i}\left(t\right)}{d_{\max}}. \end{array}$$

*d*_*i*_(*t*) is the distance between the sensor and objects that receive the ray of light. *d*_max_ is the maximum measurable distance. This equation is used to convert the value of the distance sensor to the range of 0 to 1. In addition, let **R**(*t*) = (**r**_1_(*t*), ⋯, **r**_*p*_(*t*), ⋯, **r**_|*R*(*t*)|_(*t*)) be the set of individuals of *r*_*p*_(*t*) ∈ ℝ^*N*_*r*_^, which is the vector of the distance sensor inputs in the *p*-th controller. The distance sensors are used only to calculate the fitness and not the controller input.

#### 2.2.4. Object Height Sensor

Height sensors are installed in the objects to be grasped. *h*_*p*_(*t*) is the height between the height sensor placed at the center of the object to be grasped by the *p*-th hand and the floor. *H*(*t*) = (*h*_1_(*t*), ⋯, *h*_*p*_(*t*), .., *h*_|*H*(*t*)|_(*t*)) is the set of values obtained using the height sensors. The height sensors are used only to calculate the fitness and not the controller input.

### 2.3. Controller

We use an RNN as a controller. The RNN outputs target angles of the motor through a hidden layer. Each neuron in a hidden and output layer of the RNN is updated as follows:

(4) $$\begin{array}{c} y_{i}\left(t\right)=\tanh\left(y_{i}\left(t-1\right)+\tau \underset{j\in J}{\sum }w_{ij}y_{i}\left(t-1\right)\right). \end{array}$$

Here, *y*_*i*_(*t*) is the output value of neuron *i* at step *t*. *τ* is the learning rate of the RNN. For simplicity, *τ* is set as 1 in this study. *w*_*ji*_ is the weight of the synapse connecting neurons *j* and *i*. *J* is a set of neurons input to neuron *i*. Let **W** be the set of individuals, **w**_*p*_, which is the vector of weights in the *p*-th controller. This RNN consists of an input layer, a hidden layer, and an output layer. We define *G*_RNN_ as a function that summarizes the RNN constructed from these layers for the individuals. **Y**(*t*) = (**y**_1_(*t*), ⋯, **y**_*p*_(*t*), ⋯, **y**_|*Y*(*t*)|_(*t*)), which represents the set of vectors of neurons in the output layer for the individuals, **y**_*p*_(*t*), is defined as follows:

(5) $$\begin{array}{c} Y\left(t\right)=G_{\text{RNN}}\left(Y\left(t-1\right),x\left(t\right),C\left(t-1\right),W\right). \end{array}$$

**Y**(*t*) is transformed into Θ(*t*) = (**θ**_1_(*t*), ⋯, **θ**_*p*_(*t*), ⋯, **θ**_|Θ(*t*)|_(*t*)), which is the set of target angle vectors for the individuals, **θ**_*p*_(*t*) ∈ ℝ^*N*_**θ**_^, by applying the function *G*_TA_ to derive the target angles.

(6) $$\begin{array}{c} \Theta \left(t\right)=G_{\text{TA}}\left(Y\left(t\right)\right). \end{array}$$

The controller derives the target values of the joint angles from the input values from the sensors, as indicated in Equations (5) and (6), and it is represented by the function *G*_CL_.

(7) $$\begin{array}{c} \Theta \left(t\right)=G_{\text{CL}}\left(Y\left(t-1\right),x\left(t\right),C\left(t-1\right),W\right). \end{array}$$

### 2.4. Fitness Functions and Tasks

In addition to the environment for autonomous robots to learn the pickup tasks, as described in the previous study [15–17], we add an environment for MPH control learning by using the teacher data of grasping and opening motions to enable the adaptive grasping of objects and realization of opening and closing motions at arbitrary times. In other words, we implement environment 1 to realize myoelectric control learning and environment 2 to realize object grasp learning. In each environment, a *T*_gen_-step process is implemented, and for each step, the fitness, which is a measure of the controller performance, is calculated using the fitness function. A higher fitness corresponds to a higher performance of the controller. Let *S*_*e*,*o*_ be a function that represents the computation in the simulation for the *e*-th environment involving object *o*. If a vector representing the target values of the joint angle of the *p*-th individual is input, the vector of the touch sensor and distance sensor and value obtained using the height sensor for that individual are output. If a set of target values for the joint angle of the individual is input, the set of values for the touch sensors, distance sensors, and height sensors for the individual are output, as follows:

(8) $$\begin{array}{c} S_{e,o}\left(\theta_{p}\left(t\right)\right)=\left(c_{p}\left(t\right),r_{p}\left(t\right),h_{p}\left(t\right)\right),\\ S_{e,o}\left(\Theta \left(t\right)\right)=\left(C\left(t\right),R\left(t\right),H\left(t\right)\right). \end{array}$$

In environment 2, when multiple types of grasping objects are to be learned, we run simulations for each object and sum the fitness. Then, let *O* be the set of the objects to be learned, and *o* be the element of set *O*. In environment 1, we do not employ an object to be learned. Consequently, *o* is ∅, and the subscript *o* of *S*_1,*o*_ is ignored to yield *S*_1_. If *o* is not ∅ in environment 1, *S*_1,*o*_ does not exist.

*(1) Environment 1*.The grasping and opening motions are learned according to myoelectricity. We set the total controllable joint angle as an index for the grasping and opening motions. For example, if the total value is zero for the opening motion, the total joint angle is close to zero for the opening motion, and the total joint angle is close to the maximum total angle for the grasping motion. Furthermore, the proposed method switches between grasping and opening motions. The proposed method efficiently learns the opening and closing motions by gradually accelerating the cycle of switching between the grasping and opening motions. The fitness function is set such that the fitness is higher when the total joint angle of the hand is similar to the input myoelectric motion. In addition, a hyperbolic tangent is adopted to prevent the fitness from becoming sparse.

The fitness function of *p*-th individuals in environment 1 at step *t* can be defined as follows:

(9) $$\begin{array}{c} f_{p}^{1}\left(\phi \left(t\right),S_{1}\left(\theta_{p}\left(t\right)\right)\right)=\left\{\begin{array}{cc} \frac{\tanh\left(\left(\theta_{\text{sum}}-\theta_{\text{grasp}}\right)/\omega_{\theta }\right)-\tanh\left(-\theta_{\text{grasp}}/\omega_{\theta }\right)}{\tanh\left(\left(\theta_{\max}-\theta_{\text{grasp}}\right)/\omega_{\theta }\right)-\tanh\left(-\left(\theta_{\text{grasp}}/\omega_{\theta }\right)\right)}, & \text{if }\phi \left(t\right)\text{ is grasping},\\ \frac{-\tanh\left(\left(\theta_{\text{sum}}-\theta_{\text{grasp}}\right)/\omega_{\theta }\right)+\tanh\left(\left(\theta_{\max}-\theta_{\text{grasp}}\right)/\omega_{\theta }\right)}{\tanh\left(\left(\theta_{\max}-\theta_{\text{grasp}}\right)/\omega_{\theta }\right)-\tanh\left(-\left(\theta_{\text{grasp}}/\omega_{\theta }\right)\right)}, & \text{if }\phi \left(t\right)\text{ is opening}. \end{array}\right. \end{array}$$

Here, *ϕ*(*t*) is the motion when **emg**(*t*) is measured. *θ*_max_ is the sum of the maximum joint angles of the hand, *θ*_grasp_ is the sum of the target angles for the grasping posture, *θ*_sum_ is the sum of the current joint angles, and *ω*_*θ*_ is the weight to adjust the gradient of the function.

*(2) Environment 2*. We perform both object grasp learning in addition to myoelectric control learning, as there is a possibility of forgetting the myoelectric control when only object grasp learning is performed. The design of the fitness function for object grasp learning is performed with reference to a previous study [17]. We use the values provided by the distance, touch, and height sensors to evaluate the ability of the hand to reach the object, contact the object after reaching the object, and pickup performance, respectively. The object pickup task is considered because the grasp stability can be evaluated by examining whether the hand can grasp the object, and this information can be easily obtained from the height sensor.

When the object *o* is learned, the fitness function of the *p*-th individual in environment 2 at step *t* can be expressed as

(10) $$\begin{array}{c} f_{p,o}^{2-1}\left(\phi \left(t\right),S_{2,o}\left(\theta_{p}\left(t\right)\right)\right)=\left\{\begin{array}{cc} \frac{\tanh\left(\left(\theta_{\text{sum}}-\theta_{\text{grasp}}\right)/\omega_{\theta }\right)-\tanh\left(-\theta_{\text{grasp}}/\omega_{\theta }\right)}{\tanh\left(\left(\theta_{\max}-\theta_{\text{grasp}}\right)/\omega_{\theta }\right)-\tanh\left(-\left(\theta_{\text{grasp}}/\omega_{\theta }\right)\right)}, & \text{if }\phi \left(t\right)\text{ is grasping},\\ \frac{3.5\left(-\tanh\left(\left(\theta_{\text{sum}}-\theta_{\text{grasp}}\right)/\omega_{\theta }\right)+\tanh\left(\left(\theta_{\max}-\theta_{\text{grasp}}\right)/\omega_{\theta }\right)\right)}{\tanh\left(\left(\theta_{\max}-\theta_{\text{grasp}}\right)/\omega_{\theta }\right)-\tanh\left(-\left(\theta_{\text{grasp}}/\omega_{\theta }\right)\right)}, & \text{if }\phi \left(t\right)\text{ is opening}, \end{array}\right. \end{array}$$

(11) $$\begin{array}{c} f_{p,o}^{2-2}\left(\phi \left(t\right),S_{2,o}\left(\theta_{p}\left(t\right)\right)\right)=\left\{\begin{array}{cc} 10h_{p}\left(t\right)+\overset{N_{c}}{\underset{i=1}{\sum }}c_{i}\left(t\right)+\overset{N_{r}}{\underset{i=1}{\sum }}r_{i}\left(t\right), & \text{if }\phi \left(t\right)\text{ is grasping},\\ 10h_{p}\left(t\right)-\overset{N_{c}}{\underset{i=1}{\sum }}c_{i}\left(t\right)+\overset{N_{r}}{\underset{i=1}{\sum }}r_{i}\left(t\right), & \text{if }\phi \left(t\right)\text{ is opening}, \end{array}\right. \end{array}$$

(12) $$\begin{array}{c} \left.f_{p,o}^{2}\left(\phi \left(t\right),S_{2,o}\left(\theta_{p}\left(t\right)\right)\right)\right)=f_{p,o}^{2-1}\left(\phi \left(t\right),S_{2,o}\left(\theta_{p}\left(t\right)\right)\right)+f_{p,o}^{2-2}\left(\phi \left(t\right),S_{2,o}\left(\theta_{p}\left(t\right)\right)\right). \end{array}$$

Subsequently, the fitness of the *p*-th individual in each generation *f*_*p*_(*t*) can be calculated as follows.

(13) $$\begin{array}{c} \begin{array}{c} f_{p}\left(t\right)=\underset{e\in E}{\sum }\underset{o\in O}{\sum }G_{\text{FIT}}\left(\phi \left(t\right),S_{e,o}\left(\theta_{p}\left(t\right)\right)\right)\\ =f_{p}^{1}\left(\phi \left(t\right),S_{1}\left(\theta_{p}\left(t\right)\right)\right)+\underset{o\in O}{\sum }f_{p,o}^{2-1}\left(\phi \left(t\right),S_{2,o}\left(\theta_{p}\left(t\right)\right)\right). \end{array} \end{array}$$

Here, *G*_FIT_ is the fitness calculation function. In addition, we can use the set as input from Equations (9) to (13). Therefore, *F*, which is the sum of the fitness values of the individuals after a simulation involving *T*_gen_ steps, can be defined as follows:

(14) $$\begin{array}{c} \begin{array}{c} F=\underset{e\in E}{\sum }\underset{o\in O}{\sum }\underset{t\in T_{\text{gen}}}{\sum }G_{\text{FIT}}\left(\phi \left(t\right),S_{e,o}\left(\Theta \left(t\right)\right)\right)\\ =\underset{e\in E}{\sum }\underset{o\in O}{\sum }\underset{t\in T_{\text{gen}}}{\sum }G_{\text{FIT}}\left(\phi \left(t\right),C\left(t\right),R\left(t\right),H\left(t\right)\right). \end{array} \end{array}$$

### 2.5. Learning Methods

By simulating the controllers with the set of weight vectors **W** for *T*_gen_ steps in all tasks and environments, we can obtain the set of fitness values, *F*, which is a measure of the performance of each controller. On the basis of this set, we can generate a new set of weight vectors **W**′ from **W** with the learning function *G*_L_, as follows.

(15) $$\begin{array}{c} W^{\prime }=G_{\text{L}}\left(F,W\right). \end{array}$$

**W**′ represent the candidate solutions generated from **W** to increase the fitness. By repeating this process, we identify the parameters that increase the fitness. Evolutionary computation, which is a population-based metaheuristics technique, is used as the learning scheme.

## 3. Experiments

We created a controller for a multi-DOF MPH in Unity which is a physical simulation framework by using the EMG measured in advance and verified the effectiveness of the controller. In our experiments, we aimed to verify the performance of our method and its versatility regardless of the person to whom it is applied. This verification is essential to confirm whether the designed controller can be controlled using unlearned myoelectricity. Furthermore, we conducted two comparative analyses. In Comparison 1, we compared our method with the conventional method and verified its versatility for object shapes and sizes. In Comparison 2, we compared the hand motions seen in the grasp classification and our method. Consequently, we verified the versatility for object shapes that are difficult to reproduce on the physics engine and their effectiveness for tasks other than the pickup task. The learning conditions are described in the following sections.

### 3.1. Morphology and Parameters

#### 3.1.1. Anthropomorphic Robotic Hand Model

We established an anthropomorphic robotic hand model, as shown in Figure S1, by using primitive objects of Unity, which is a game engine, and used this model as the agent. The DOF of this agent was based on the kinematics model reported by Miyata et al. [18]. The dimensions were based on the average values of the Japanese hand dimensions data provided by AIST [19]. The joint positions were based on the functional length of the phalanges, as reported by Hamilton and Dunsmuir [20]. The range of motion of the fingers was set by referring to two existing studies [21, 22]. The mass of the prosthetic hand was set as 520 g, based on the results of a previous study [23] that showed that the upper limit of the mass of a prosthetic hand was approximately 500 g. The joint ranges of motion are presented in Table 1(a), and the dimensions are listed in Table 1(b). The other parameters are listed in Table 1(c).

#### 3.1.2. Target Angle Derivation Function

The target angles of the joints were derived by applying the target angle derivation function to the outputs of the controller, as follows:

(16) $$\begin{array}{c} \Theta \left(t\right)=G_{\text{TA}}\left(Y\left(t\right)\right)=\left(0.5Y\left(t\right)+0.5\right)\left(\Theta_{\max}-\Theta_{\min}\right). \end{array}$$

Θ_max_and Θ_min_ represent sets of the maximum and minimum angles of the joints, respectively.

#### 3.1.3. Motor

We assumed an experimental environment in which the angular velocity of the joints was controlled by proportional control. In this case, the angular velocity vector **v**_*p*_(*t*) of the joint angle of the *p*-th individual can be described using the weight *ω*_*v*_:

(17) $$\begin{array}{c} v_{p}\left(t\right)=\omega_{v}\left(\theta_{p}\left(t\right)-\theta_{c}\right). \end{array}$$

**θ**_*c*_ is the vector of the current joint angles.

#### 3.1.4. Input from EMG Sensor

We used an integrated electromyogram (iEMG) as the input from the EMG sensor. The iEMG was calculated using the function *G*_iEMG_, as follows:

(18) $$\begin{array}{c} G_{\text{iEMG}}\left(emg\left(t\right)\right)=\overset{T_{\text{EMG}}}{\underset{k=0}{\sum }}\left|emg\left(t-k\right)\right|, \end{array}$$

(19) $$\begin{array}{c} iemg\left(t\right)=G_{\text{iEMG}}\left(emg\left(t\right)\right). \end{array}$$

*T*_EMG_ is the number of samples. Subsequently, we normalized *iemg*(*t*) from −1 to 1 by using the normalization function *G*_NM_:

(20) $$\begin{array}{c} G_{\text{NM}}\left(iemg\left(t\right)\right)=\frac{2\cdot iemg\left(t\right)}{{emg}_{\max}\cdot T_{\text{EMG}}}-1, \end{array}$$

(21) $$\begin{array}{c} {iemg}_{\text{nm}}\left(t\right)=G_{\text{NM}}\left(iemg\left(t\right)\right). \end{array}$$

In general, the magnitude of the EMG signal varies from person to person. Therefore, we scaled the iEMG input signal from -1 to 1 by using scale transformation function *G*_SC_, with *iemg*_max_ being the maximum value for each channel.

(22) $$\begin{array}{c} G_{\text{SC}}\left({iemg}_{\text{nm}}\left(t\right)\right)=\left\{\begin{array}{cc} 1, & \text{if }{iemg}_{\text{nm}}\left(t\right)>{iemg}_{\max},\\ \left({iemg}_{\text{nm}}\left(t\right)+1\right)\cdot \frac{2}{1+{iemg}_{\max}}-1, & \text{otherwise}. \end{array}\right. \end{array}$$

Using Equations (18) to (22), the following expression can be obtained:

(23) $$\begin{array}{c} G_{\text{FE}}\left(emg\left(t\right)\right)=G_{\text{SC}}\left(G_{\text{NM}}\left(G_{\text{iEMG}}\left(emg\left(t\right)\right)\right)\right). \end{array}$$

### 3.2. Learning Conditions

The following learning conditions were adopted in this work.

We simulated *P* = 50 agents for *T*_gen_ = 1200 steps. As described in the previous subsection, one task was conducted in environment 1 and two tasks were conducted in environment 2 for each generation to obtain the total value of the fitness function. The weights of the RNN of the controller were updated based on this value. This process was repeated for *N*_gen_ = 250 generations (participants A–E) or *N*_gen_ = 300 generations (participant F). Subsequently, we evaluated the optimized agents by comparing the associated findings with those reported previously and those obtained using conventional methods. In this subsection, we describe the EMG input conditions, tasks, and learning techniques.

#### 3.2.1. EMG Input Condition

*(1) EMG Measurement*. EMG data were measured from five able-bodied adult participants and a child participant with forearm deficiency, with the permission of the ethics committee of the University of Electro-Communication (permit No. 10006(5)). For EMG measurement, three channels of electrodes [24] were used for participants A-E and two channels for participant F. The number of channels was reduced in participant F because there is less space to place electrodes on the residual limb. Consequently, the process was repeated for more number of generations for participant F in comparison with the other participants. The sensors were attached, as shown in Figure S2. For participants A-E, ch1 was attached to around the palmaris longus, flexor carpi ulnaris, and flexor digitorum superficialis; ch2 was attached to around the extensor digitorum, and extensor carpi radialis brevis; and ch3 was attached to around the flexor carpi radialis and the pronator teres. For participant F, a child with an upper limb deficiency, ch1 was attached to the flexor and ch2 to the extensor. The participants were asked to sit down and repeat the grasping and opening motions as instructed. The grasping here refers to a fist-clenching motion without holding any object. The measurer visually confirmed that each movement was performed as instructed before taking the measurements. The EMG was converted to the iEMG according to Equations (18) and (19). We measured 200 iEMGs simultaneously for the two movements of grasping (flexion contraction) and opening (extensor contraction). These values were measured ten times alternately from grasping. The other measurement conditions are presented in Table 1(d). A 50 Hz high-pass filter and a notch filter were used to mitigate 50 Hz electromagnetic noise generated by commercial power supplies in eastern Japan. These two filters were used together because the notch filter alone was not sufficient to remove this noise.

**Figure 2:**
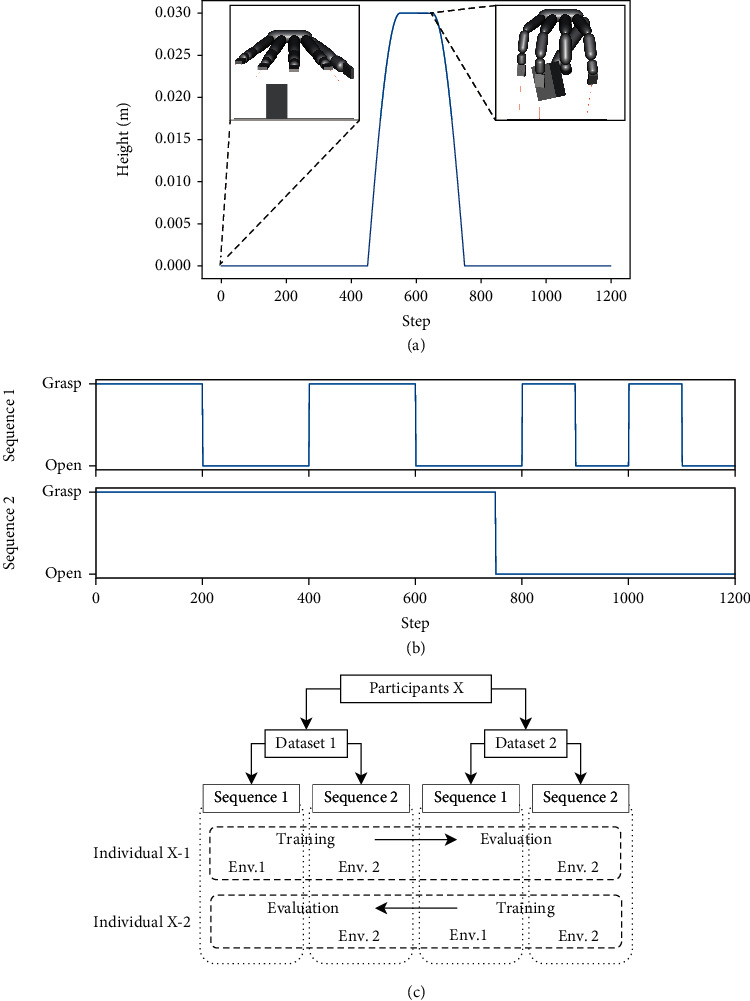
(a) Trajectory of the hand and screenshots of the hand at the 0-th step and the highest position. The scale of the screenshots is the same. (b) Input sequence of EMG signals. (c) Relationships among the datasets, learning and evaluation schemes, and individuals in the experiments. Two datasets are created for each participant. Each dataset is transformed into two sequences. Sequences 1 and 2 refer to environments 1 and 2, respectively. The individuals with the highest fitness among the controllers learned from datasets 1 and 2 for participant X are known as individual X-1 and X-2, respectively. Sequence 2 is used for the evaluation.

*(2) Input Sequence for the EMG Sensor*. We switched the input sequence of the iEMG signals depending on the environment because different environments require different movements to accomplish the relevant tasks. We divided the 3200 measured iEMG signals into two datasets with 1600 iEMG signals per channel without changing the order and trained two models with the data for each participant. The data from each channel were converted to be in the sequence shown in Figure 2(b), without changing the order of the data for each hand movement. Sequences 1 and 2 were used as the inputs for environments 1 and 2, respectively. The first and second halves of the dataset for each participant were referred to as datasets 1 and 2, respectively.

#### 3.2.2. Tasks

The agents performed three tasks in two environments. The obtained total value of the fitness was used for optimization. This section describes the tasks and environments.

*(1) Environment 1*. The agents performed the tasks of opening and closing the hand. Specifically, the agents performed the tasks of grasping and opening the hand by using the EMG of the grasping and opening motions, respectively. However, at this time, the agent's controller was not provided any information regarding the hand motion at the time of EMG measurement.

*(2) Environment 2*. The agents attempted to pick up a cuboid and a sphere object. The dimensions are presented as Cuboid0 and Sphere2 in Table S2. The EMG obtained from the grasping motion of the user was used as an input to the EMG sensor during the pickup motion. After picking up the object, the EMG obtained from the opening motion of the user was input, and the opening motion is performed. Next, the object was released. The agent did not lift the object using the controller, and the hand model was programmed to rise automatically from the 451st step to the 750th step and then return to the original position. The trajectory of the hand is shown in Figure 2(a). We adopted a cuboid instead of a cube as the target object of the pickup task to prevent the agent from falling into a local optimum when picking up a cube, which requires contacting the floor with the tip of the hand.

The parameters of the fitness functions in each environment were set as follows: the sum of the maximum joint angles of the hand is *θ*_max_ = 1440, sum of the target angle for the grasping posture is *θ*_grasp_ = 720, and weight for adjusting the gradient of the function is *ω*_*θ*_ = 400.

#### 3.2.3. Learning

To set the learning function *G*_L_, we used the covariance matrix adaptation evolution strategy (CMA-ES) [25], which is an evolutionary computation method that performs a multipoint search by considering a normal distribution. The parameters recommended by Hansen and Auger [26] were adopted.

### 3.3. Experimental Condition: Performance Evaluation of Optimized Agents

The relationships among the datasets, learning and evaluation schemes, and individuals are shown in Figure 2(c). Two datasets were created for each participant. Each dataset was transformed into two sequences. Sequences 1 and 2 corresponded to environments 1 and 2, respectively. The performance of the optimized agents was evaluated using the dataset that was not used for training (evaluation dataset). The optimized controllers were selected from the population after training by extracting the ones with the highest fitness using the evaluation dataset as shown in Table S1. The individual with the highest fitness among the controllers learned from dataset 1 for participant X is called individual X-1 and for dataset 2, individual X-2. Sequence 2 is used for evaluation.

In the evaluation, we considered an object to be grasped successfully if it did not touch the floor between the 551st and 650th steps and when the hand was stagnant in space, as shown in Figure 2(a), which corresponds to the trajectory of the hand in a 1200-step simulation. If the object was released after 1200 steps, the opening motion was considered to have been performed. Cuboid0 and Sphere2 objects, which were used in training, were used in the evaluation. Furthermore, we verified whether the opening motion could be realized by checking the transition of the average value of the joint angle and change when the input EMG was switched.

### 3.4. Comparison 1: Comparison with the Conventional Method

In the experiment in subsection 3.3, we verified the performance of our method and its versatility regardless of the person to whom it is applied. However, it is necessary to verify the method's versatility for different shapes and sizes of grasped objects and its superiority over conventional approaches. The adaptive grasping method described in Section 1 is challenging to reproduce on a physics engine, and the software approach requires more input signals, making it difficult to make a fair comparison. Here, the approach by Hoshigawa et al. is easy to compare with the proposed method.

#### 3.4.1. Conventional Method: Optimized Hand Shape

Hoshigawa et al. developed a simple prosthetic hand with 2-DOF controlled by pattern recognition [27]. The prosthetic hand is capable of flexion and extension of the four fingers and thumb. The shape of the fingers has been optimized by pick-and-place experiments using actual devices so that the fingers can grasp tools that are expected to be frequently used in activities of daily living (ADL). This method is one of the hardware-based approaches described in Section 1, and as with adaptive grasping, the shape of the hardware is limited.

Artificial neural network is used as a pattern recognition method to distinguish between grasping and opening motions, and this control method is also used for the method of Hoshigawa et al. in this comparison experiment. For training, we used dataset 2 (sequence 2 of participant C). The other parameters were set as summarized in Table 2.

#### 3.4.2. Comparison Conditions

We performed the object pickup tasks as described in subsection 3.3 and compared the performance values. The individual C-1 model was used. For both types of methods, sequence 2 translated from dataset 2 of participant C was used for the comparison. The following characteristics of the object were changed:

- Object shape
- Orientation of the object relative to the hand

**Figure 3:**
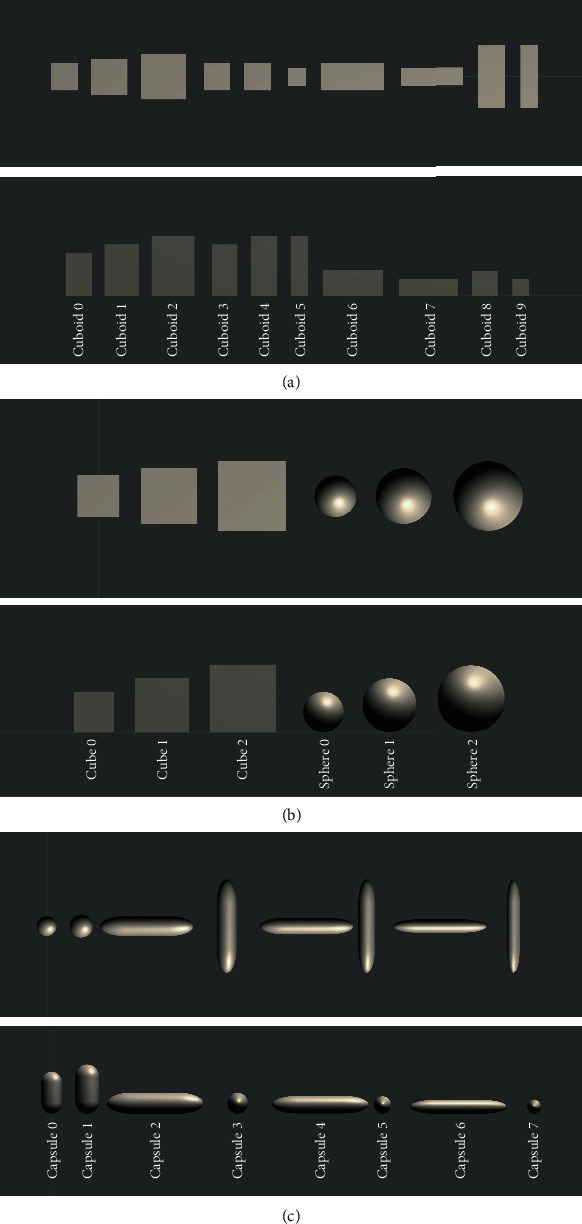
Object shapes. Top of each diagram: top view, bottom: front view. (a) Cuboids, (b) cubes and spheres, and (c) capsules.

These aspects were changed sequentially, and we verified whether the agent could grasp the object in each condition. We considered an object to be grasped successfully if it did not touch the floor between the 551st and 650th steps and when the hand was stagnant in space as in the subsection 3.3. The appearances of the shapes are shown in Figure 3.

In addition, when a user grasps an object using an MPH,

- the position of the object relative to the hand

is a factor controlled by the user. Therefore, we considered that the user can grasp the object if the position of the object is changed sequentially and the pickup achievement condition is satisfied in any of the ranges. As shown in Figures S3a, S3c, and S3e, we set 100 object positions during training.

### 3.5. Comparison 2: Comparison with the Grasping Classification

In the previous subsection, we verified the generalizability to various objects, but there is a limit to the object shapes that the physics engine can verify. Therefore, the versatility of complex object shapes must be evaluated by grasping simple objects verified by the physics engine. The study of the taxonomy of grasps, which explores the versatility of hand forms, can be evaluated in this manner. Grasping classification is a classification of static motions in the human hand and is used for training goals in rehabilitation and the design of robotic hands and prostheses. In the research on prosthetic hands, the ability to realize many grasping motions is one of the performance evaluation criteria, and it can be considered one of the evaluation methods of versatility for grasping objects. Typical examples are Kamakura's grasping taxonomy [10], which classifies hand motions observed in daily life, and Cutkosky's grasping taxonomy [29], which classifies hand motions observed by workers in machining factories. In this study, we compared these two classifications with the hand movements observed by our method. These two taxonomies have different task domains: one caters to fundamental grasping tasks, whereas the other caters to grasping movements that are more complex and diverse. The proposed model is evaluated using these two taxonomies to verify its applicability to different grasping conditions and modalities.

The individual C-1 model, which had the highest fitness, as indicated in Table S1, was considered. sequence 2 translated from dataset 2 of participant C and was used for the comparison. The comparison method involved changing the shape of the primitive object in Unity (cube, sphere, and capsule), have the agent grasp the object, and record the grasping motion if it matched any of the grasping motion classifications.

## 4. Results

In this section, we describe the results of the experiments and comparisons.

### 4.1. Performance Evaluation of the Optimized Agents

**Figure 4:**
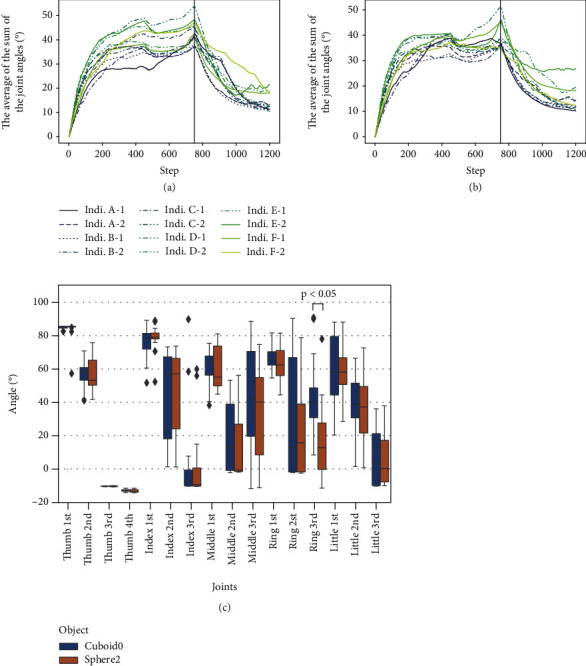
Changes in the mean values of the joint angles when the agent picks up (a) Cuboid0 and (b) Sphere2. The vertical line shows the time at which the EMG input switches from the EMG of the grasping movement to that of the opening movement. Indi. is an abbreviation for individual. (c) The figure shows the average joint angles for all individuals during the 551-651 steps when the hand is stagnant after grasping an object in the air. The blue graph shows Cuboid0, and the orange graph shows Sphere2. The rhombic points represent outliers. The joint angles are slightly out of the upper and lower limits because the body objects are pushed out by the objects when they come into contact with each other, and the relative values between the body objects shift slightly.

**Figure 5:**
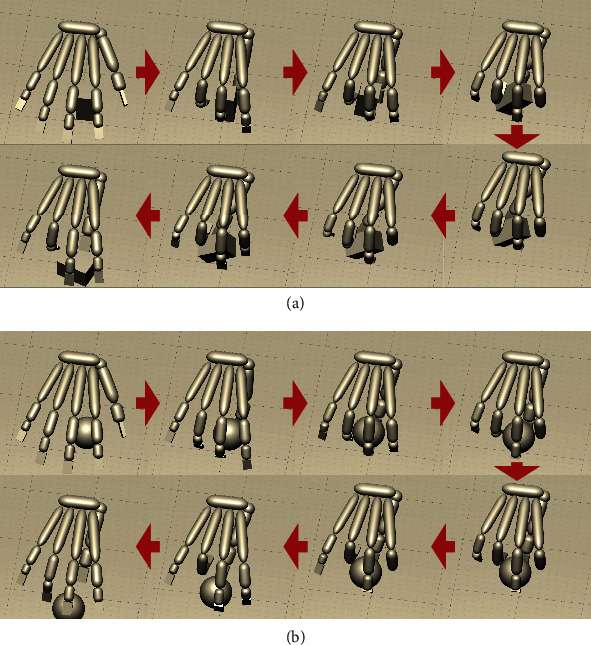
The figures show how the Individual F-1 grasps (a) Cuboid0 and (b) Sphere2, respectively. The red arrows indicate the passage of time. The following links will take you to the videos. (a) https://youtu.be/6oYrVZ_Bjis and (b) https://youtu.be/XHXHHmOMrUQ.

This subsection presents the results of the performance evaluation of the agents trained using the EMG signals of five able-bodied adult participants (A–E) and a child participant (F) with forearm deficiency.  Table 3 presents the results of examining whether the agent trained on each dataset could pick up and release the object. Figures 4(a) and 4(b) show the average joint angles when picking up Cuboid0 and Sphere2, respectively.  Figure 4(c) shows the average joint angles for all individuals during the 551-651 steps when the hand is stagnant after grasping an object in the air. The table indicates that Cuboid0 and Sphere2 could be lifted and closing and opening motions could be performed according to the myoelectricity data for all participants. The average angle at the 750th step, when the myoelectricity during the grasping motion switched to that during the opening motion, was significantly larger than the average angle at the 1200th step (Wilcoxon's rank-sum test, Cuboid0: *p* = 0.0011, Sphere2: *p* = 0.0011). Figures 5(a) and 5(b) show how the individual F-1 grasps Cuboid0 and Sphere2.

### 4.2. Comparison with the Conventional Method

Table 4 presents the results of comparing the performance of the conventional and proposed methods in multiple object pickup tasks. Checkmarks (✓) indicate objects that were successfully grasped in the pickup task. The proposed method enabled the successful grasping of all the considered objects.

### 4.3. Comparison with Taxonomy of Grasps

#### 4.3.1. Comparison with Cutkosky's Taxonomy of Manufacturing Grasps

**Figure 6:**
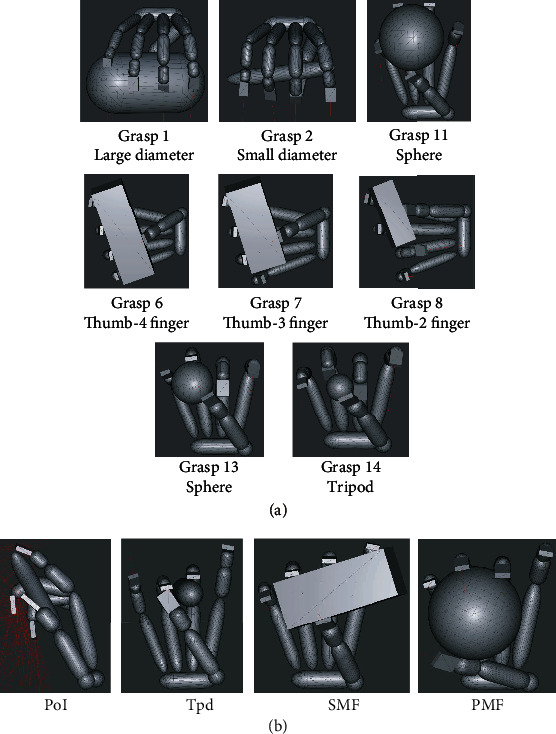
(a) Matching of the grasping motions with Cutkosky's classification of grasping motion. The numbers represent the classification number of the grasp. (b) Matching of the grasping motions with Kamakura et al.'s grasping motion classification. The labels indicate the classification number of the grasp.

The results of the comparison with Cutkosky's taxonomy of manufacturing grasps and the matched movements are shown in Figure 6(a). Eight out of the 16 movements matched.

#### 4.3.2. Comparison with Kamakura et al.'s Grasping Motion Classification

The results of the comparison with Kamakura et al.'s grasping motion classification and matched movements are shown in Figure 6(b). Four out of the 14 movements matched.

## 5. Discussion

This section discusses the results of the verification.

### 5.1. Results of the Learning Process

Successful opening and closing motions could be performed according to the EMG. Moreover, Cuboid0 and Sphere2, which were the objects used for learning, could be lifted using the EMG data of the six participants including a child participant with forearm deficiency. These findings indicated that the proposed method is suitable for a wide variety of individuals.

### 5.2. Comparison with the Conventional Method

Compared with the existing method, the proposed method enables the robotic hand to successfully grasp all the considered objects (Table 4). In our validation, we used objects with a flat surface (Cuboid0) and curved surface (Sphere2) for training. Therefore, the grasping strategies learned using these two objects are likely effective against unlearned shapes (capsules, in this work) and objects with different sizes.

Massera et al. suggested that “certain behavioral strategies might be effective for a large variety of objects, and the limited differences in terms of shape and size of the objects to be grasped should not necessarily impact the rules that regulate the robot/environmental interactions.” [15].

The results of this study support this suggestion.

### 5.3. The Grasping Posture Observed in This Study

Figure 4 shows that the angle of the first joint of the thumb is the largest when grasping an object, indicating that the thumb faces the middle finger and ring finger during grasping. From Figure 5, we can see that precision grasping is used. In general, precision grasping is performed with the thumb facing the index finger or the index finger and the middle finger [10, 29]. According to the study by Cotugno et al., the range of motion of the thumb has been reported to be biased towards forming a precision grasp (opposing the index finger or the index and middle fingers) in many robot hands [30]. This indicates that this grasping method is the standard posture adopted by robotic hands. However, this behavior was not observed this time.

This discrepancy can be attributed to two causes. Kuo et al. defined the functional workspace of the precision thumbâ€“finger grasp in their study. They defined it as the range of all possible positions in which the thumb tip and each fingertip can simultaneously contact each other. They found that the functional workspace of thumb-directed opposition is the largest among the four fingers [31]. In other words, generating a stable motion trajectory with the thumb facing the index finger is difficult. To prevent this complexity, human anatomical structures such as muscles, tendons, and ligaments related to the thumb contribute to stabilizing the thumb in humans [32]. However, the proposed method imitates only the joint structure of the human hand. Thus, the proposed model may have adopted a strategy to stabilize the thumb by maintaining the first joint at the maximum angle instead of stabilizing the thumb with muscle tendons.

Second, the precision grasp with the mother and index finger facing each other was not optimal for the pickup task because it is a grasping strategy specialized for object manipulation, as indicated by the size of the functional workspace in this case. However, the proposed method does not have a task related to object manipulation. Thus, it may be that the thumb can perform the pickup task more stably when it is facing the middle or ring fingers than when it is facing the index finger. Kong et al. found that the contribution of each finger to the grasping force increases in the following order: middle finger, ring finger, index finger, and little finger [33]. If we assume that the contribution to the grasping force affects the stability of grasping an object, this finding validates the above discussion.

Therefore, it can be considered that the proposed method expresses a grasping motion that is appropriate for the hand form and task. Furthermore, although the precise grasping with the thumb and the index finger facing each other is typical in robots, the proposed method enables us to discover the possibility of a new grasping motion that is rarely seen in humans.

### 5.4. Comparison with the Grasping Motion Classification: Results of Matching Conducted Using Two Taxonomies

As shown in Figure 6, eight out of 16 grasping motions were matched with Cutkosky's taxonomy, and four out of 14 motions were matched with Kamakura et al.'s taxonomy. Based on these results, we discuss the limitations of our method. Three significant differences exist between the taxonomies of Cutkosky and Kamakura et al. These are discussed below.

The first significant difference between the two taxonomies is that Cutkosky's taxonomy is based on the skeleton of the hand, whereas Kamakura et al.'s taxonomy is based on the palmar contact distribution. In other words, to realize a grasping motion with the grasping posture classified by Kamakura et al., in most cases, the palm must establish contact with the object. However, in our proposed method, we focus on reproducing the kinematic model of the hand, and not the palm. Therefore, reproducing the grasping motion that requires the palm is difficult.

Second, Cutkosky's grasping taxonomy is intended only for one-handed tasks in contrast to the two-handed movements considered by Kamakura et al. The task set in this study is also a one-handed task that does not consider cooperative behavior, which can be seen in the results.

The third significant difference is the complexity of the tools used: Cutkosky considers tools from the factory; most are simple objects such as cylinders, spheres, and disks. In contrast, Kamakura et al. consider several objects with complex shapes such as keys, scissors, and chopsticks, in addition to spheres. However, the behavior of the structural complexity of the objects used for training does not seem to affect the behavior. One of the hypotheses of 3D object recognition is that 3D objects are recognized by an arrangement of simple geometric elements called “geons” [34]. In a study measuring Japanese monkey brain cells, it has also been suggested that the shape of the hand may be created in advance by referring to primitive geometric features (such as flat, round, and elongated) that are common to similar objects [35–37]. Thus, learning to grasp objects with primitive geometric features may potentially enable the grasping of complex objects. The experimental results show that learning to grasp only primitive objects (Cuboid0 and Sphere2) enabled the grasping of unlearned shapes and objects. This supports the hypothesis that the structural complexity of the object used for learning does not affect the learned behavior of the model.

In summary, the comparison of grasping motion classifications revealed issues that we need to address to realize a variety of hand movements: reproduction of grasping using the palm, realization of cooperative behaviors, and a search for optimal and efficient primitive objects for use in training.

## 6. Conclusion

By combining evolutionary robotics with the control of a myoelectric prosthetic hand, we have achieved adaptive grasping using myoelectricity. The proposed model predicts the appropriate joint motion based on the information of the contact state between the hand and grasped object obtained from the touch sensor. As a result of training the agents of the 16-DOF robot hand with the myoelectric signals of six participants, including one child with an upper limb deficiency, all the agents could accomplish the pickup tasks. In addition, regardless of the participants, the agents performed the pickup tasks using a similar posture for a given object, indicating that the hand was optimized by evolutionary computation to a posture that prevents the object from being dropped. This result indicates that the proposed method can adaptively grasp objects with simple myoelectric control using a multi-DOF myoelectric prosthetic hand, regardless of the participants.

These results show that an approach that considers both hardware and software is effective for “interpolating the human grasp function,” which has not previously been realized. In grasping experiments with objects of different shapes and sizes, the agent grasped objects of unlearned shapes and sizes. This result supports Massera's observation that certain behavioral strategies are effective for grasping a wide variety of objects and that constraints on the shape and size of the object do not necessarily affect the rules that control the interaction with the hand. This result contributes to the development of versatile multi-DOF robot hands. Most notably, we argue for the importance of a hardware/software fusion approach to determine the hand motion considering the interaction between the object and the hand as a solution to “ interpolate the subconsciously performed human adaptive grasp function,” which researchers have left to the prosthetic hand users. To the best of our knowledge, this is the first effective solution considering both hardware and software for a myoelectric prosthetic hand. The effectiveness of our study has also been validated using EMG data from a child with congenital forearm defects. This result shows the applicability of our method to people with upper limb deficiencies.

However, our method also has a few limitations. The proposed method is only intended to supplement the adaptive capability of grasping in the pickup task. In practice, it is desirable to be able to grasp an object while considering subsequent and cooperative actions. Moreover, the switching of actions must be studied to effectively apply the method to tasks other than the pickup task. In addition, the grasping posture was restricted in this experiment to ensure the stability of thumb control. In practice, the control of the human hand is stabilized by anatomical structures, especially the muscles, tendons, and ligaments. Thus, an additional mechanism that reproduces these structures in hardware must be developed and applied to this research. Therefore, as future works, we plan to work on switching the actions to tasks other than the pickup task and verifying the grasping motion's effectiveness when the anatomical structures pointed out in this study are considered.

## Figures and Tables

**Table:** (a) Joint range of motion

	Thumb				Other fingers		
	1st	2nd	3rd	4th	1st	2nd	3rd
Max [°]	80	60	90	90	90	100	90
Min [°]	-10	-10	0	0	-10	0	-45

**Table:** (b) Dimensions of a five-finger robotic hand model

Finger	Part	Height [mm]	*ϕ* [mm]	Mass [kg]
Thumb	Metacarpal	62.7	19.1	0.05
	Proximal phalanx	30.2	19.1	0.01
	Distal phalanx	23.2	16.6	0.01

Index finger	Metacarpal	89.7	17.2	0.07
	Proximal phalanx	40	17.2	0.02
	Middle phalanx	22.6	14.9	0.01
	Distal phalanx	17.4	13.3	0.01

Middle finger	Metacarpal	87.3	17.3	0.07
	Proximal phalanx	45.4	17.3	0.02
	Middle phalanx	25.6	15.2	0.01
	Distal phalanx	19.7	14.4	0.01

Ring finger	Metacarpal	82.5	16.2	0.07
	Proximal phalanx	43.2	16.2	0.02
	Middle phalanx	24.4	14.2	0.01
	Distal phalanx	18.8	13.8	0.01

Little finger	Metacarpal	75.5	14.3	0.06
	Proximal phalanx	33.4	14.3	0.01
	Middle phalanx	16.7	12.8	0.01
	Distal phalanx	16.7	11.4	0.01

Trapezium	19.1		19.1	0.01
Carpals	55.5		19.1	0.02

			Sum	0.52

**Table:** (c) Parameters for a five-finger robotic hand

Part	Parameter	Value
Joint	Number	16
Motor	*ω*_*v*_	2
EMG sensor	Number [ch]	3
	Sampling points (*T*_EMG_)	5
Touch sensor	Number	5
	Placement	On each distal phalanx
Distance sensor	*d*_max_	0.08 (m)
Controller	Number of neurons in the input layer	8
	Number of neurons in the hidden layer	30
	Number of neurons in the output layer	16

**Table:** (d) Measurement condition of the EMG

Sampling frequency (Hz)	2667.683
Quantization bit rate (bit)	14
Filter (hardware)	· 50 Hz notch filter
	· Bandpass filter with a
	1–1000 Hz bandwidth
Filter (software)	50 Hz high pass filter
Potential range (V)	-2.5 to 2.5

**Table 2:** Parameters of the conventional recognition method.

Recognition method *G*_PR_	Artificial neural network
Number of neurons (input layer)	3
Number of neurons (hidden layer)	24
Number of neurons (output layer)	2
Learning method *G*_L_	Adam [28]

**Table 3:** Results of examining whether the agent can lift the object by using the EMG data of each participant.

	Cuboid0	Sphere2
Success rate (%)	100 (*n* = 12)	100 (*n* = 12)

**Table 4:** Results of the object grasping experiment. Checkmarks (✓) indicate objects that were successfully grasped in the pickup task.

	Cuboid0	Cuboid1	Cuboid2	Cuboid3	Cuboid4	Cuboid5	Cuboid6	Cuboid7	Cuboid8	Cuboid9	Cube0	Cube1	Cube2
Proposed method	✓	✓	✓	✓	✓	✓	✓	✓	✓	✓	✓	✓	✓
Conventional method	✓	✓	✓	✓	✓	✓	✓	✓			✓	✓	✓

	Sphere0	Sphere1	Sphere2										
Proposed method	✓	✓	✓										
Conventional method													

	Capsule0	Capsule1	Capsule2	Capsule3	Capsule4	Capsule5	Capsule6	Capsule7					
Proposed method	✓	✓	✓	✓	✓	✓	✓	✓					
Conventional method			✓		✓								

## Data Availability

The data used to support the findings of this study are available from the corresponding author upon reasonable request.

## Supplementary Materials

Supplementary Materials Table S1: final fitness of the learned controllers. Table S2: dimensions of the objects to be grasped. Figure S1: 16-DOF anthropomorphic robotic hand model. Figure S2: sensor layout of participants. Figure S3: range of object placement.
