# Supplementary material for: Coevolution of Myoelectric Hand Control under the Tactile Interaction among Fingers and Objects
Source: Cyborg Bionic Syst. 2022 Nov 16;2022:9861875. doi: 10.34133/2022/9861875 (PMC9691400; doi:10.34133/2022/9861875)

Cyborg and Bionic Systems
A SCIENCE PARTNER JOURNAL

Permission for Photographic/ Illustration Use

I hereby grant to Beijing Institute of Technology Press ("BITP") a perpetual and irrevocable non-exclusive right to use and authorize others to use the photo/illustration/image/figure below (the "Material(s)") in connection with an article on

Co-Evolution of Myoelectric Hand Control
Under the Tactile Interaction Among Fingers and Objects

(the "Article") to be published and distributed by BITP and its licensees/ assigns in an issue of the journal *Cyborg and Bionic Systems*, in any and all media in which the Article and issue may be published or distributed, and for any later use by BITP of that Article in any format or medium now known or hereafter developed, including Web-based publishing.

I warrant that I am the owner and/or authorized representative of the owner of the copyright in the Material(s) and that I have the authority to grant BITP the permission and rights granted herein. I further warrant that the Material(s) and use thereof does not and will not violate the copyright or other personal or proprietary right of any person.

Authorization

Full Name: Yuki Kuroda

Signature: Yuki Kuroda Digitally signed by Yuki Kuroda Date: 2022.06.01 21:49:45 +09'00'

* Attach images on page 2 before signing

Date: 06/01/2022

Images covered by this permission:

Filename: 5Finger_PickUpArea_back.PNG

Name to appear in credit line: Yuki Kuroda

INSERT THUMBNAIL IMAGE OF MATERIAL(S)

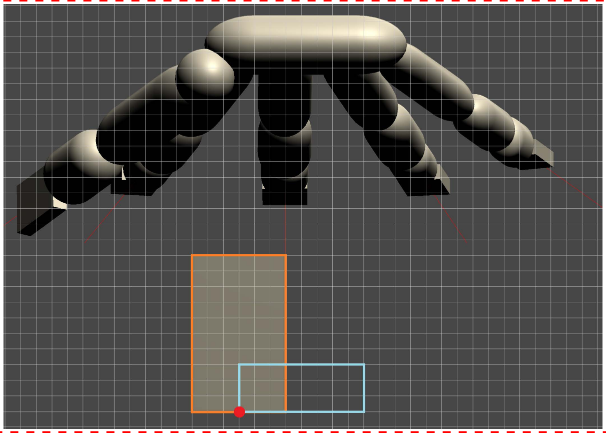

Supplement: Supplementary Materials — Table S1: final fitness of the learned controllers. Table S2: dimensions of the objects to be grasped. Figure S1: 16-DOF anthropomorphic robotic hand model. Figure S2: sensor layout of participants. Figure S3: range of object placement. [file 9861875.f1.zip › image_permission_Cyborg_5Finger_PickUpArea_back.pdf]
